# Understanding Perceptions and Practices for Zambian Adults in Western Province at Risk for Hypertension: An Exploratory Descriptive Study

**DOI:** 10.5539/gjhs.v8n2p248

**Published:** 2015-07-08

**Authors:** Nelly D. Oelke, Kathy L. Rush, Fastone M. Goma, Jessica Barker, Patricia Marck, Chloe Pedersen

**Affiliations:** 1 School of Nursing, Faculty of Health and Social Development, University of British Columbia, Kelowna, Canada; 2 Department of Physiological Sciences, University of Zambia School of Medicine, Lusaka, Zambia; 3 University of Cape Town, Cape Town, South Africa

**Keywords:** adult, hypertension, health practices, mixed methods, non-communicable diseases, chronic disease management, public health policy, community engagement, Zambia

## Abstract

Hypertension is an important public health issue in Zambia. Despite the need for early detection, treatment, and ongoing monitoring, there is little documented research on hypertension in Zambia. The study aims were to: 1) better understand risk factors for hypertension in urban and rural communities in Mongu and Limulunga Districts, Western Province; 2) identify current health practices for hypertension and prevention in these communities; and 3) explore intersections between culture and hypertension perceptions and practices for study participants. A mixed methods approach was used; 203 adults completed surveys including demographics, anthropometric measures, blood pressure (BP), physicial activity, diet, and salt intake at five health check stations. Two focus groups were conducted with rural and urban community members to better understand their perspectives on hypertension. The prevalence of hypertension was 32.8% for survey participants. A further 24.6% had pre-hypertension. The mean total weight of salt added to food was nearly double the WHO recommendation with women adding significantly more salt to food than men. Significant differences in waist circumference were observed between men and women with men at low risk and women at substantialy high risk. In focus groups, participants cited westernized diets, lack of physical activity, stress, psychological factors, and urbanization as causative factors for hypertension. Participants lacked understanding of BP medications, healthy lifestyles, adherence to treatment, and ongoing monitoring. Focus group participants mentioned challenges in obtaining treatment for hypertension and desired to be active contributors in creating solutions. They recommended that government priorize hypertension initiatives that increase access to health education to reduce risk, enhance early detection, and support lifestyle changes and medication adherence. Our findings suggest that policy-makers need to engage communities more effectively to develop successful public health strategies to prevent, detect, and manage hypertension in Western Province, Zambia, particularly in rural areas.

## 1. Introduction

The prevalence of Non-Communicable Diseases (NCDs), such as hypertension (HTN), in Sub-Saharan Africa (SSA) is a major public health concern (Thorogood, Myles, Hundt, & Tollman, 2007; van de Vijver et al., 2013; Yach, Hawkes, Gould, & Hofman, 2004). Uncontrolled HTN may result in systemic physiological complications such as cardiovascular and renal disease and has become a leading cause of morbidity and mortality in SSA (Thorogood et al., 2007; Twagirumukiza et al., 2011; World Health Organization [WHO], 2007). In particular, Zambia is faced with a growing problem of NCDs and is significantly impacted by HTN (Republic of Zambia Ministry of Health, 2011), resulting in negative consequences for individuals, communities, and the health system. The Zambian health system faces challenges in health service delivery including inadequate funding, critical shortages and inequitable distribution of health workers, erratic supply of essential medicines and medical supplies, and insufficient, as well as inequitable, distribution of health infrastructure, equipment, and transportation (Republic of Zambia Ministry of Health, 2011). The potential effects of uncontrolled HTN may place a further burden on Zambia’s already strained health system. It is, therefore, imperative that HTN become a priority for health researchers, the Zambian government, and stakeholders within the healthcare system.

### 1.1 Background

The prevalence of HTN varies across African countries and among regions within countries. HTN rates ranging from 19% to as high as 40% were found in countries that participated in the WHO-STEPS surveys (2003-2009) (van de Vijver et al., 2013). A systematic review conducted to assess the burden of HTN in SSA found a median prevalence of HTN of 29%, and a random effects model combined prevalence across all of the 33 studies at 30% (Ataklte et al., 2015). Within a Southern African context, prevalence studies have also shown similar findings. In a population-based nationwide cross-sectional survey in Malawi, Msyamboza et al. (2011) found a HTN prevalence of 33%. Goma et al. (2011) also reported a HTN prevalence of 34.8% in an urban area of Zambia. A HTN rate of 32% was found in a study conducted in another urban area of Zambia (Siziya, Rudatsikira, Babaniyi, Songolo, Mulenga, & Muula, 2012). Research conducted in two rural districts of Zambia, found a 26% rate of HTN among its participants in Kaoma and 30% in Kasama (Mulenga et al., 2013). Age was a significant factor associated with HTN where increased age was correlated with higher rates of HTN (Dewhurst et al., 2013; Goma et al., 2011; Mulenga et al., 2013; Siziya et al., 2012; van de Vijver et al., 2013). Gender was found to be associated with HTN in some studies but not in others. Goma et al. (2011) and Pobee (1993) found higher rates of HTN in male participants, while Dewhurst et al. (2013) found HTN was more common among female participants although their study was conducted with older adults only. On the other hand, no significant difference was found in HTN rates between males and females in other reports and studies (Mulenga et al., 2013; Siziya et al., 2012; van de Vijver et al., 2013). Other risk factors for HTN also varied. HTN was associated with tobacco use (Goma et al., 2011; Mulenga et al., 2013; van de Vijver et al., 2013), frequent alcohol use, lack of physical activity, (Goma et al., 2011; van de Vijver et al., 2013), high sodium intake (van de Vijver et al., 2013), low fruit and vegetable consumption (van de Vijver et al., 2013), increased BMI (Goma et al., 2011; Mulenga et al., 2013; Siziya et al., 2012; van de Vijver et al., 2013), increased heart rate (Mulenga et al., 2013), high cholesterol, and impaired glucose levels (Goma et al., 2011).

Socio-cultural factors have been shown to play a pivotal role in influencing perceptions and beliefs about HTN (Maseko, Majane, Milne, Norton, & Woodiwiss, 2006). Poverty, health literacy, and demands of family and work life have been shown to affect a person’s ability to adhere to a medical treatment plan (Kagee, Le Roux, & Dick, 2007). Diet has been reported as a major contributing factor to HTN (Maseko et al., 2006). A study to determine the relationship between sodium and blood pressure (BP) found that hypertensive participants had higher urinary sodium excretion levels which were indicative of higher sodium intake (Azinge, Sofola, & Silva, 2011). Changing culturally engrained behaviours, such as diet and lifestyle, can be difficult and take time (Risenga, Botha, & Tjallinks, 2007). Cultural influences on HTN prevention and management practices, including risk factor management, remain largely under-researched. Additionally, there has been a lack of research, particularly qualitative research to understand cultural influences on risk factors and health practices specific to HTN in Zambia. Examining cultural perspectives and norms will assist policy-makers and health care providers in better understanding how to manage HTN and encourage healthier lifestyles for Zambian people (Kagee et al., 2007).

Hypertension remains under-researched in Zambia, despite the necessity for early detection, treatment, and monitoring. With only three quantitative studies located on HTN in Zambia and only one of them conducted with rural populations, further investigation of the local context for HTN prevention and management in Zambia is required to strategically plan and deliver cost-effective services in a resource constrained environment. Our qualitative study fills an important gap in our current knowledge about the Western Province’s context in relation to the growing incidence of this chronic disease.

### 1.2 Study Aims

The objectives for this study were: 1) to better understand risk factors for hypertension in urban and rural communities in Mongu and Limulunga Districts, Zambia; 2) to identify current health practices for hypertension management in these communities and 3) to explore how cultural beliefs intersect with HTN perceptions and health practices.

## 2. Method

A mixed-method design was used for this study to enhance and enrich data collected on HTN in Zambia (Loiselle, Profetto-Mcgrath, Polit, & Tetano Beck, 2011). Mixed-method designs mitigate against problems associated with the use of a single method (Creswell, 2014) and assist researchers to elicit the meaning of quantitative results (Creswell, 2014; Loiselle et al., 2011). Furthermore, mixed-method designs permit the researcher to gain a better understanding of the need for interventions (Creswell, 2014). To achieve a comprehensive understanding of HTN prevention and management, quantitative data informed qualitative data collection and qualitative data were used to better understand the “why” of the quantitative data. Triangulation allowed the research team to better understand and interpret data and improve credibility (Loiselle et al., 2011).

### 2.1 Participants and Data Collection

Quantitative data were collected in one rural and one urban community in Mongu and Limulunga Districts, Western Province, Zambia. Community members were recruited via radio announcements and word of mouth. Participants were eligible if they lived in the community and were 18 years of age and older. Five “health check” stations were set up in high traffic areas such as local markets and grocery stores. Face-to-face interviews were administered after verbal consent was obtained from the eligible candidates. Anthropometric (e.g., weight, waist circumference) and BP measurements were made using standard routine procedures (WHO, n. d.a). Nursing students from Lewanika School of Nursing and the University of British Columbia, Okanagan Campus collected data. Students received prior training on taking anthropometric and BP measurements and administering surveys; all data collection was supervised by two research team members familiar with the Zambian context and with more than 40 years of collective nursing education experience. A local translator was present at each station and standardized scripts were used to provide details about the study.

A modified version of the World Health Organization (WHO) STEPwise survey for surveillance was used for quantitative data collection (WHO, n. d.b). The STEPwise tool uses a population-based approach to chronic disease risk factor surveillance and has been used in a variety of settings within SSA. As per STEPwise protocols, data were collected on demographics, BP, weight, height, body mass index (BMI), waist circumference, and self-reported health behaviours (e.g., diet). Physical activity was measured using the International Physical Activity Questionnaire (IPAQ) Short Form (Booth, 2000). Salt intake was measured by having each participant draw from a container, using hands, measuring cups, or spoons, the amount of salt they typically used at breakfast, lunch, and dinner. A total volume of added salt intake per day was calculated from these values. To determine daily salt added to food in grams, a conversion tool was used to convert the volume measurement to grams (See http://www.traditionaloven.com/culinary-arts/cooking/table-salt/convert-tea-spoon-tsp-to-gram- g.html). Once all data were collected, all those found to be hypertensive were given a referral to the weekly Medical Clinic at the local General Hospital.

Qualitative data were gathered through two focus groups; one rural and one urban focus group were conducted with community members from Mongu and Limulunga Districts. Focus groups have been shown to be particularly effective when engaging members of a diverse cultural group (Halcomb, Gholizadeh, DiGiacomo, Phillips, & Davidson, 2007). Focus groups allowed the research team to clarify meaning with participants and identify local and culturally appropriate interventions for the prevention and management of HTN (Halcomb et al., 2007). Participants were recruited using radio advertisements and word of mouth. They were eligible to participate if they were 18 years or older, not currently receiving health care for HTN, and not pregnant. Recruitment focused on community members who varied by age, gender, and rural-urban location to obtain diverse perspectives on the topic of HTN. Informed consent was obtained prior to data collection and participants completed a demographic questionnaire including age, sex, education level, marital status, occupation and work status, the number of adults living in the household, and average income. BP measurements were also completed.

A semi-structured interview guide was utilized to facilitate open discussion about underlying beliefs, concerns, and knowledge regarding HTN, BP monitoring, and alternative treatments. Participants were asked about the role of social institutions, personal experiences with the health care system, and types of health services they would find beneficial. Data were recorded digitally and field notes were documented to describe the research setting, participant behaviours and actions. Field notes were integral to the interpretive descriptive design because they allowed the researcher to guide and retrace the focus group process (Thorne, Kirkham, & Macdonald-Emes, 1997). Incentives for participating in the study included reimbursement of transport.

### 2.2 Data Analysis

All quantitative data were entered into an Excel™ database and imported into the Statistical Package for Social Sciences for Windows (SPSS)™ version 22.0 for analysis. Focus group demographic data were also entered into Excel™ and analyzed using the same program. Descriptive statistics were used to summarize demographic data and other study variables. Chi-square tests of association were used to analyze relationships between two demographic variables, age and sex, and salt and anthropometric variables (waist circumference and BMI) that were stratified according to levels of risk when compared to standardized norms. The level of significance for all analyses was set at p < .05. Focus group discussions were recorded and transcribed by local Zambian translators to ensure accuracy. Focus group data were entered into NVivo9™ qualitative software for coding. Data were thematically analyzed by one research team member using open coding, categorizing, clustering similar units of meaning, and identification of relationships and patterns between and across categories (Richards & Morse, 2012). Coding was then reviewed and discussed with one of the principal investigators and revisions made as appropriate. Research team members met frequently to debrief, discuss emerging themes, and discuss interpretation of both quantitative and qualitative data. Data collected from this study were compared to pilot research conducted previously.

### 2.3 Ethics

Ethics approval was obtained from the UBCO Behavioural Research Ethics Board (H13-00191) and the University of Zambia Biomedical Research Ethics Committee.

## 3. Results

Data were collected from March 2013–May 2013. A total of 203 adults completed interview surveys at Health Checks and 50 community members participated in two focus groups. Demographic data for survey and focus group participants are summarized in Table 1. Just over 900,000 people live in Western Province and the majority of the population is rural. Forty-eight percent of the population is male and 52% is female. The overall population in Western Province is fairly young; 47% are under 15 years of age, 50% are age 15-64, and 4% are 65 and older (Central Statistical Office, 2012).

**Table 1:** Participant demographic information

Demographic characteristics		Survey participants		Focus group participants	

		Frequency	Percent	Frequency	Percent
Age	Age group	Range-18-80;	Mean-39; SD-12.5	Range-19-85;	Mean 49.5; SD-18.7

	18-29	49	26.1	11	23.9
	30-39	55	29.3	4	8.7
	40-49	41	21.8	5	10.9
	50-59	34	18.1	12	26.1
	60+	9	4.8	14	30.4

Sex	Male	98	48.3	16	35.6
	Female	105	51.7	29	64.4

Marital Status	Never married	41	20.3	9	19.6
	Currently married	120	59.4	21	45.7
	Separated or divorced	21	10.4	8	17.4
	Widowed	20	9.9	8	17.4

Education Level	Primary school or below	66	32.6	9	19.6
	Secondary or high school completed	106	52.3	29	63.0
	College/university completed	31	15.3	8	17.4

Work	Government employee	25	12.3	3	6.7
	Non-governmental employee	23	11.3	5	11.1
	Self-employed	115	56.7	22	48.9
	Unemployed (able to work)	23	11.3	5	11.1
	Other (student, homemaker, retired, unable to work, non-paid)	17	8.4	10	22.2

Further results have been divided into three sections: current HTN status and management practices; risk factors for HTN; and cultural beliefs and HTN.

### 3.1 Current Hypertension Status and Management Practices

Participants’ BP readings were categorized into the American Society of Hypertension, International Society of Hypertension, and Eighth Joint National Committee classification of HTN (Page, 2014) including normal (<130/<80), pre-hypertension(130-139/80-89), Stage 1 (140-159/90-99) and Stage 2 (>159/>99). Overall, the prevalence of HTN from survey participants was found to be 32.8%. A further 24.6% had pre-hypertension. Of those who had hypertension, 37.5% were male and 62.5% were female. These results are presented in Table 2.

**Table 2:** Blood pressure by age and sex (% of total)

Blood pressure	18-29 years of age		30-39 years of age		40-49 years of age		50-59 years of age		60 years of age and older	

	Male	Female	Male	Female	Male	Female	Male	Female	Male	Female
**Normal (<130/<80)**	3.9	3.9	8.8	5.6	8.3	3.3	6.1	<2.5	<2.5	<2.5

**Pre-hypertension (130-139/80-89)**	3.9	3.3	2.8	<2.5	2.8	<2.5	<2.5	5.0	<2.5	--

**Stage 1 (140-159/90-99)**	<2.5	5.6	3.3	2.8	2.8	<2.5	--	<2.5	--	--

**Stage 2 (>159/>99)**	<2.5	<2.5	<2.5	<2.5	--	<2.5	--	2.8	<2.5	<2.5

**Total**	11.1	15.0	16.6	11.7	13.9	8.8	6.7	11.1	2.8	2.2

Over half of the participants (56.2%) stated they had ever had their BP checked by a doctor or healthcare worker. Of these, 49.1% were informed they had an elevated BP and 66% of them were informed of their raised BP within the previous 12 months. Thirty-nine percent of participants who were not told about their high BP were found to have Stage 1 or 2 HTN when their BP was measured at the Health Check Station. Of the individuals who had never had their BP checked, 28.6% of those had HTN. Of those participants who had ever been told they had a raised BP, 76.7% had received medications in the last two weeks. During the previous 2 weeks, only 10.3% of those participants who were found to have HTN at the Health Check Station had been treated for raised BP with medications. Many individuals with HTN were not receiving medications for their high BP.

Understanding of HTN differed between urban and rural community members. Rural members appeared to have a lower level of comprehension of HTN. However, all community members lacked an understanding about antihypertensive medications, healthy lifestyles, and the importance of continuing BP treatment. Focus group participants recognized the severity of the disease. This was particularly evident in the rural group as compared to urban participants. Rural participants used statements such as *“How are we going to live?”* (FG Participant), *“It is a serious disease”* (FG Participant), and *“many lives are lost”* (FG Participant) to describe their deep concerns about the disease.

Difficulty in accessing health care centres, insufficient equipment and understaffed clinics were cited as major barriers to treatment in both rural and urban sites. Under stocked medication was a primary reason noted for lack of adherence with treatment. Sub-optimal provision of clinical services with long wait times at health centres were described by the majority of participants. Inconsistent BP monitoring and apathetic attitudes among health care providers were also cited.

Focus group participants, particularly in the rural community, felt their opinions on HTN were not valued and they did not have a voice in this issue. They desired to be actively involved in identifying solutions for HTN prevention and management in their community. Participants suggested HTN have a priority comparable to communicable diseases such as HIV. They desired timely access to health services for prevention, treatment and ongoing monitoring of BP. Participants also expressed a desire for government funding to support antihypertensive medications and ensure a consistent available supply.

All of us here have been tested for hypertension and the government should provide drugs the way [it does for] ART [Antiretroviral Therapy] clients; the BP clients [need to] be treated the same. They should look for a Centre for BP patients and [the] Ministry should supply/provide more types of drugs to all the health facilities. (FG Participant)

Participants recommended health promotion initiatives to increase awareness of risks of HTN including lifestyle changes and the importance of following medication regimes. Furthermore, prevention education was also desired.

### 3.2 Risk Factors for Hypertension

Numerous beliefs about the causes of HTN were identified; beliefs were similar between rural and urban groups. These included: westernized diet (high intake of fat, sugar, and salt), lack of physical activity, genetics, stressful life events, psychological factors, and urbanization.

**Figure 1:**
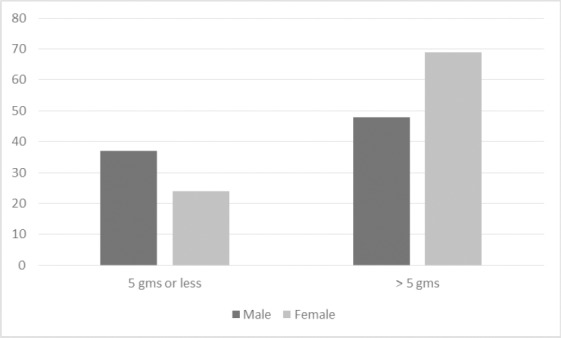
Salt intake by sex (frequency) (p<0.05)

High fat and sodium intake and low numbers of fruit and vegetable servings characterized participants’ diets. Salt was added to food at all meals, more at lunch and dinner than breakfast, with a mean total weight of salt added to food equaling 9.33 ± 10.03 grams while the WHO recommended a salt intake of 5 gms/day (WHO, 2012). Figure 1 shows salt intake by sex. A higher number of women added more salt than the recommended daily intake than men (p <0.05).

Survey participants reported eating fruit an average of 2.18 ± 2.2 days per week with a mean of 1.38 ± 1.15 servings on one of those days. Vegetables were eaten an average of 5.40 ± 2.3 days per week with a mean of 1.86 ± SD .90 servings on one of those days. A majority of the survey participants (82.3%) used vegetable oil most often in meal preparation with lard or suet, butter or ghee, margarine, and other types of fat used in descending order. On average, participants ate 1.42 ± 1.9 meals per week outside of the home.

**Figure 2:**
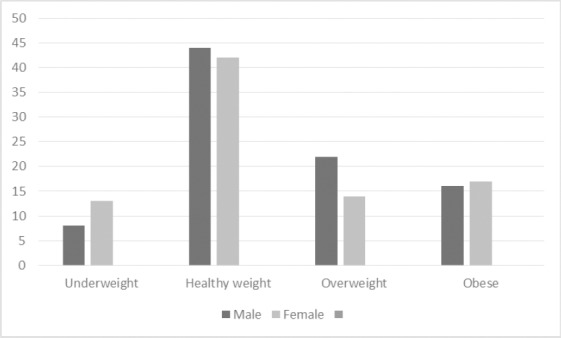
BMI by sex (frequency)

**Figure 3:**
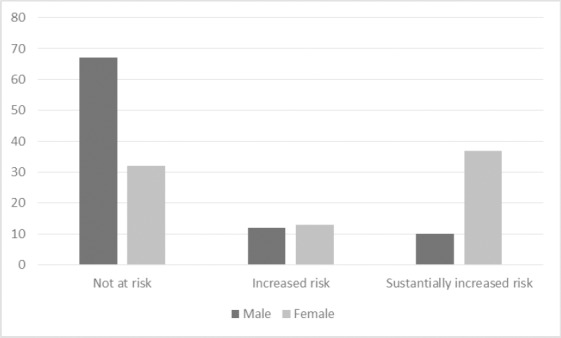
Waist circumference by sex (frequency) (p<0.01)

Weight was found to be highly variable, ranging from 29 to 118 kilograms with a mean weight of 68.97 kg (SD 17.42). BMI was calculated from participants’ weight and height measurements. Twelve percent of participants were classified as underweight (BMI of <18.5), 49% of participants were a healthy weight (BMI: 18.5-24.9), 21% were overweight (BMI 25-29.9), and 19% were obese (BMI >30). BMI by sex is presented in Figure 2. Overall there was little variation in BMI for males and females. Waist circumference ranged from 59 to 175 centimeters with a mean circumference of 88.46 (SD 18.05). As shown in Figure 3, more women than men were substantionally at risk for metabolic complications because of their waist circumference (p<0.01) as defined by the WHO (2008) (not at risk [male ≤ 94; female ≤ 80]; increased risk [male > 94; female > 80]; substantially increased risk [male > 102; female > 88]). Urban participants appeared to have a greater understanding of the connection between diet and weight and BP, as demonstrated with the statement *“if I had avoided some of the foods maybe I would not be like this”* (FG Participant).

Stressful life events were also perceived to be a cause of HTN. To some extent, community members believed BP was outside of their control. During discussion about HTN prevention, a participant from the urban group stated, *“It’s difficult to avoid [hypertension] because by our nature you can’t avoid thinking, can’t avoid certain eventualities that occurs in families, things like death, illnesses. Those are the thing that brings [increased] BP, so the question of avoiding [it] is difficult. You can’t avoid [it] because naturally we live with them”* (FG Participant).

Focus group participants identified the impact of HTN on their health from symptom disturbances to death. These included symptoms such as visual disturbances, palpitations, fatigue, stress, and generalized illness. Some community members recognized cardiovascular and neurological complications, including the risk of stroke. Finally, several community members talked about death as a possibility of uncontrolled and untreated HTN.

### 3.3 Cultural Beliefs and Hypertension

Two areas of cultural beliefs were recurrently identified in participants’ comments on the prevention and management of HTN: beliefs about western and traditional medicine for the treatment of HTN and beliefs about exercise and physical activity. Participants were divided in their opinions about the effectiveness of western medicine or herbal remedies for treating HTN. While a few participants from the urban group admitted they used herbal medicines for BP management, most agreed that *“the best is to go to the hospital and see the specialist people”* (FG Participant). In contrast, rural members used a wide diversity of alternative medicines and herbs as the preferred treatment for HTN.

Cultural practices surrounding exercise and physical activity was a topic of conversation among urban community members but was not discussed in the rural focus group. Urban participants agreed that while exercise may prevent HTN, one might be ridiculed for exercising in public. *“If you happen to be found doing exercises, playing games with children, people will say you are running mad”* (FG Participant). Furthermore, admission to recreation centres was too expensive for most participants.

## 4. Discussion

This research focused on further understanding HTN in urban and rural communities in Western Province, Zambia using both quantitative and qualitative approaches. Understanding the current state of HTN in these communities, potential risk factors, current health services and prevention as well as the influence of cultural practices on the understanding of HTN and its treatment is essential in the development of policies and changes to practice.

The problem of HTN in SSA clearly emerged in the current findings with 32.8% of study participants with a systolic BP of 140 or greater and/or a diastolic BP of 90 or greater. Rates of increased BP in Mongu and Limulunga Districts were similar to those found in other SSA studies with reported rates ranging from 29-33% (Ataklte et al., 2015; Msyamboza et al., 2011). HTN rates were also similar to those found in Lusaka, Zambia (34.8%) (Goma e et al., 2011) and Kitwe, Zambia (32%) (Siziya et al., 2012), but higher than rates found in two other rural communities in Zambia (26%; 30%) (Mulenga et al., 2013). Our findings also identified a further 24.6% of individuals with pre-hypertension, suggesting that over half of our sample (57.4%) were at risk for developing complications due to high BP. Furthermore, only 10.3% of participants with a high BP were receiving medication for the same. Of the participants who had high BP in the current study, approximately two thirds had had their BP checked by a health care provider. Many of the participants with a high BP were not previously aware of their high BP. Although we did not ask if participants were aware of a HTN diagnosis it is clear that a large percentage (37.5%) were not as they had never had their BP checked by a health care provider. Our study found HTN rates were higher in women than in men. Most studies in SSA and Zambia have not shown a difference between HTN rates for men and women (Mulenga et al., 2013; Siziya et al., 2012; van de Vijver et al., 2013). The higher prevalence of hypertension in women may reflect their higher estimated salt intake and increased waist circumference compared to men.

This is one of the few studies to estimate daily salt intake and showed an average of 9.33 gms/day of salt added to an individual’s diet, approximately double the WHO’s recommended daily salt intake of 5 gms/day (WHO, 2012). This exceeds salt intake in some high income countries such as that of 8.1 gms/day in the United Kingdom (Department of Health, Government of the United Kingdom, 2012). The problem of high salt intake may be exercabated in African countries due to their lack of understanding of the link between salt intake and HTN, its use in food preservation, and the palatability it adds to an otherwise bland diet (van de Vijver et al., 2013). Access and affordability of fruit and vegetables is likely an important factor impacting high dietary salt intake. Furthermore, the amount of salt added to food was higher among women than men. Perhaps women are able to better estimate the amounts of salt added to foods given that they would have the major responsibility for preparing food for their families. Ways of decreasing sodium intake and increasing vegetable and fruit intake must be explored and embedded in health promotion messages in the community.

Participants described the impact of urbanization and westernized influence on themselves and their communities as coming “from the outside.” Both rural and urban focus group participants identified such influences as genetically modified and westernized foods (high sugar, fat, and sodium) as impacting the prevalence of HTN. Van de Vijver et al. (2013) identified urbanization as a contributing demographic change impacting increased HTN. However, additional information is required to fully comprehend the impact of westernization and urbanization on HTN and health practices in these Zambian communities.

A westernized view of exercise and physical activity did not seem to resonate with our Zambian participants. Despite urban participants’ understanding of the importance of physical activity in the prevention of HTN, exercising was not a common practice and would be viewed negatively by other community members. Physical activity and exercise were not even discussed by community members in the rural area. This may be a reflection of the fact that rural life comprises more physical activity such as farming and walking. Further exploration into the meaning of exercise and physical activity for these community members, both urban and rural, may assist with identifying culturally acceptable approaches to physical activity.

The belief that a person could not control whether they would get HTN was a concern raised by community members in focus groups. Participants noted “thinking too much” as a major cause of HTN. Many believed the underlying reasons for HTN such as death in the family, illness for one’s self or family members, and stress were outside of their control. Increasing knowledge and understanding of HTN as a chronic disease with modifiable risk factors would assist in empowering individuals with HTN to make changes in their lifestyle to improve quality of life and decrease morbidity and mortality related to HTN.

Knowledge gaps were prevalent among community participants especially related to the etiology of HTN and medication management. Although participants were aware of the serious health risk that HTN posed to their community, they lacked understanding of prevention strategies, healthy lifestyles, and treatment options. Such factors as high cost, lack of resources, and side effects may be possible reasons for the lack of pharmacological use in the treatment and management of HTN as found in other studies (Gama et al., 2013; Kagee et al., 2007) but this study extended the reasons to include a lack of understanding of the etiology of HTN. Providing linkages between etiological factors and lifestyle changes may ensure better outcomes for people living with HTN. Providing information about different treatment modalities and how each works in combination would likely increase treatment adherence. The impact of health literacy on adherence to a treatment plan also needs to be considered (Kagee et al., 2007).

Participants’ desired change in health services for the prevention, diagnosis, and management of HTN. Given the insufficiency of infrastructure and health promotion and disease prevention strategies and the lack of government funding for HTN management, national policies for HTN as a government priority are required. Furthermore, movement towards comprehensive and integrated management of diseases rather than the current disease specific approach will facilitate better outcomes for these communities. Participants recommended HTN be approached in the same manner as communicable diseases (e.g., HIV). This is an important finding in the study. There appears to be recognition that NCDs require as much attention as communicable diseases given the rising rates of NCDs in African countries and the changing demographics of an aging population (van de Vijver et al., 2013). Perhaps too, participants have witnessed better care approaches to addressing communicable disease and feel that these approaches hold promise for addressing HTN. Lessons learned from HIV that can be applied to HTN and public health initiatives for the prevention of HTN would be beneficial to raise awareness and promote healthier lifestyles. Equipping health centres with equipment for monitoring BP and a consistent drug supply chain to treat and manage HTN, recurrent issues in the literature (Gama et al., 2013; Kagee et al., 2007), are desperately needed.

These findings also suggest services in community health centres in the Districts of Mongu and Limulunga need to be dedicated to the diagnosis and treatment of NCDs. One such initiative that has been successful in many underserved communities is the integration of community health workers (CHWs) for NCDs (Tulenko, 2013). The inclusion of CHWs in health teams allows frequent service-user interaction at the community level, which improves adherence, follow-up and psychosocial support (WHO, 2008). The effective use of CHWs could be an innovative approach to expand the depth and breadth of NCD prevention and management at a community level. In 2010, Zambia was in favour of a national CHW strategy; however, inconsistent support, fragmented funding, and role ambiguity presented barriers to the full integration of this public health strategy (Tulenko, 2013). Furthermore, the impacts of CHW integration were not quantified. Therefore, more research needs to be conducted to determine the impact and cost-effectiveness of CHWs.

Task shifting is an option to consider given the health human resource crisis in many SSA countries. Some countries, including Zambia, have adopted the Nurse Initiated and Managed Antiretroviral Therapy (NIMART) program, called the HIV Nurse Practitioner (HNP) program, whereby nurses are trained in specific HIV service tasks including the initiation and management of antiretroviral therapy. NIMART has been a critical strategy in scaling up the provision of HIV services, particularly in more remote and underserved areas (WHO, 2008). A similar strategy could be undertaken to improve access and management of NCDs in these Zambian communities. Elements of the NIMART program could be used and nurses trained to provide comprehensive HTN management including; recommending pharmacologic treatment regimens, clinical monitoring, and managing side effects of medications (Zuber et al., 2014). The use of CHWs for education and prevention for NCDs, particularly in rural areas, is another form of task shifting that could be promoted.

### 4.1 Limitations

The generalizability of these results to other communities and areas of Zambia will need to be carefully considered. Given the variability in HTN rates and risk factors, generalizability of results to other areas must be undertaken with caution. In addition, our sample size for quantitative data is not large (n=203). Furthermore, participants may have self-selected to be involved in the study or recruitment strategies may have unintentionally biased those who responded. Our sample includes few older adults (n=9), although this is reflective of the small percentage of this segment of the population in Western Province, Zambia. Further research will be required to capture data on this segment of the population.

Fieldwork in rural Zambia with limited resources presented some challenges. Quantitative data were collected by trained fourth year nursing students supervised by University of British Columbia, Okanagan Campus nursing faculty. Even so, there may have been some variation in the way in which data were collected. Completing surveys in busy environments along with participants’ time constraints made completion of the lengthy survey more difficult. Language barriers and literacy level were mitigated by the use of translators. Survey data were based on participants’ self-report and therefore not as reliable as direct observation, although anthropometric measures and BP were collected. Finally, multiple data collectors using different BP monitors may have contributed to inconsistent readings across participants.

An additional survey (IPAQ Short Form [Booth, 2000]) was also used in an attempt to gather information on physical activity. The concept of physical activity may have been misunderstood, as some responses obtained from the survey were implausible (e.g., exercising eight days per week).

Only one focus group was held per site (but with large representation); hence, generalizability of the data may be more limited. Fewer than two focus groups for each participant type, such as urban vs. rural, leaves some uncertainty that emergent themes accurately depicted the various perceptives of the larger population (Morgan, 1995). Therefore, additional focus groups should be conducted in future research to ensure accuracy and saturation is achieved.

Large numbers of participants in the focus groups may have impacted some participants from sharing during the discussion. Group dynamics and cultural beliefs may influence the comfort level of participants and willingness to disclose information (Halcomb et al., 2007). Nevertheless, all participants shared at one point during discussion and participants who were quieter appeared to agree with the dominant speakers as indicated by their body language.

## 5. Conclusion

HTN is a significant concern in Western Province of Zambia. HTN prevalence was greater in women than men. Women had higher estimated salt intakes and waist circumferences, two factors putting them at risk. Overall, participants are keen to learn more about HTN prevention and management and strongly expressed the need for support to reduce the health challenges resulting from HTN in their community. There is an important role for policy-makers in shaping policies for NCDs such as HTN. Development of an effective locally adapted public health strategy to prevent, detect, and manage HTN is essential.
